# Abnormal Resting-State Activities and Functional Connectivities of the Anterior and the Posterior Cortexes in Medication-Naïve Patients with Obsessive-Compulsive Disorder

**DOI:** 10.1371/journal.pone.0067478

**Published:** 2013-06-28

**Authors:** Yuqi Cheng, Jian Xu, Binbin Nie, Chunrong Luo, Tao Yang, Haijun Li, Jin Lu, Lin Xu, Baoci Shan, Xiufeng Xu

**Affiliations:** 1 Department of Psychiatry, The First Affiliated Hospital of Kunming Medical University, Kunming, Yunnan, PRC; 2 Department of Internal Medicine, The First Affiliated Hospital of Kunming Medical University, Kunming, Yunnan, PRC; 3 Key Laboratory of Nuclear Analysis Techniques, Institute of High Energy Physics, Chinese Academy of Sciences, Beijing, PRC; 4 Key Laboratory of Animal Models and Human Disease Mechanisms, Chinese Academy of Sciences & Yunnan Province, Kunming Institute of Zoology, Kunming, Yunnan, PRC; 5 Magnetic Resonance Imaging Center, The First Hospital of Kunming City, Kunming, Yunnan, PRC; Institute of Psychiatry at the Federal University of Rio de Janeiro, Brazil

## Abstract

**Background:**

Obsessive-compulsive disorder (OCD) is a mental illness characterized by the loss of control. Because the cingulate cortex is believed to be important in executive functions, such as inhibition, we used functional magnetic resonance imaging (fMRI) techniques to examine whether and how activity and functional connectivity (FC) of the cingulate cortex were altered in drug-naïve OCD patients.

**Methods:**

Twenty-three medication-naïve OCD patients and 23 well-matched healthy controls received fMRI scans in a resting state. Functional connectivities of the anterior cingulate (ACC) and the posterior cingulate (PCC) to the whole brain were analyzed using correlation analyses based on regions of interest (ROI) identified by the fractional amplitude of low-frequency fluctuation (fALFF). Independent Component Analysis (ICA) was used to identify the resting-state sub-networks.

**Results:**

fALFF analysis found that regional activity was increased in the ACC and decreased in the PCC in OCD patients when compared to controls. FC of the ACC and the PCC also showed different patterns. The ACC and the PCC were found to belong to different resting-state sub-networks in ICA analysis and showed abnormal FC, as well as contrasting correlations with the severity of OCD symptoms.

**Conclusions:**

Activity of the ACC and the PCC were increased and decreased, respectively, in the medication-naïve OCD patients compared to controls. Different patterns in FC were also found between the ACC and the PCC with respect to these two groups. These findings implied that the cardinal feature of OCD, the loss of control, may be attributed to abnormal activities and FC of the ACC and the PCC.

## Introduction

Two core characteristic symptoms of obsessive-compulsive disorder (OCD), recurrent intrusive thoughts (obsessions) and repetitive behaviors or mental acts (compulsions), are thought to be related to dysfunction in executive function processes [1]. The impairment of executive functions, including planning [1], decision-making [2], response inhibition [3] and action monitoring [4], are all found in OCD patients. Neuroimaging and lesion studies have demonstrated that executive functions are most often associated with particular regions of the prefrontal cortex [5], especially the anterior cingulate cortex (ACC), the dorsolateral prefrontal cortex (DLPFC) and the orbitofrontal cortex (OFC). Abnormal (usually, increased) activity of the cingulate region, which often correlates with the severity of obsessive symptoms [2], has been observed in OCD patients and has been normalized after successful treatment [6]. Hyperactivation of neural circuits, such as the cortico-striatal circuit (CSTC) found in the prefrontal cortex (PFC), the ACC and the striatum, has also been implicated in OCD [7], [8]. The cortico-striatal interaction has been the focus of most research on the CSTC model [9], [10]. According to the newly revised CSTC model for OCD postulated by some researchers, many other brain circuits and regions may also be involved in OCD [11], [12]. A functional connectivity study revealed significantly enhanced connectivity between the dorsal ACC and the DLPFC, supporting a theory of abnormal error processing and introducing a cortico-cortical interaction that may affect decision-making in OCD [13]. In these studies, an abnormal functional connection between the ACC and the PFC was found in OCD. Both the ACC and the PFC are important to executive functions. Neuroimaging research has shown that the ACC is activated both during error trials and during correct, incongruent trials [14], [15]. The PFC is involved in the control of other forms of higher-order cognition. Damage to the PFC impairs decision-making. Additionally, the PFC is involved in task preparation and switching between different tasks. Therefore, it is thought to be involved in many aspects of executive control over thoughts and actions. Importantly, researchers have also found increased PFC activation in control trials following conflict-and-error trials and correlations between the activation of the ACC in conflict-and-error trials and of the PFC on the subsequent control trials, supporting the presence of a “conflict-control loop.” The ACC is proposed to detect the presence of conflict and to alert the PFC to resolve the conflict [16].

Vogt et al. concluded that the cingulate cortex as a whole can be divided into an anterior region (ACC), concerned with executive control, particularly of emotion-related processes, and a posterior region (PCC) that is specialized for evaluative and monitoring functions [17]. However, the function of the PCC in OCD patients has been less studied. A recent meta-analysis revealed that the PCC preferentially responded to positive rewards, whereas the ACC and the lateral PFC selectively responded to negative rewards [18]. However, the functional coupling of the PCC with the medial PFC, the DLPFC and the ACC was also found in normal adults [19]. Thus, it is possible that the ACC and the PCC may show distinct functions and be responsible for different pathologies in OCD.

These results reveal the complex and unbalanced functions of the ACC and the PCC in OCD. However, most of these results come from task-relevant design studies. The resting-state activities or connectivities of the cingulate cortexes to other brain regions in OCD remain unclear. Traditionally, resting-state functional connectivity (FC) patterns are examined by correlating the resting fMRI time series of a single-seed voxel against the time series of all other voxels, resulting in a functional connectivity map (FCMap). Using these region of interest (ROI) methods, the strong correlations between selected regions to other regions can be found. However, the information provided by an FCMap is limited to the network associated with the selected seed voxel. The patterns of organization for whole brain networks cannot be found. In contrast, model-free methods, such as independent component analysis (ICA) [20], [21], enable the exploration of spatial and temporal activation patterns without the need for defining a specific model. ICA methods have the advantage of identifying sub-networks with subtly different spatiotemporal patterns, without specific ROI restriction. Previous studies using ICA have revealed the ACC and the PCC as the main components of different resting-state sub-networks (sub-RSNs) in healthy subjects [22], individuals with autism spectrum disorder [23], and individuals with social anxiety disorder [24]. Thus, it is possible that the ACC and the PCC might have different functional organizations or in the different sub-RSNs and control independent functions separately in OCD. To the best of our knowledge, studies of the characteristics of the sub-RSNs in OCD have been very limited.

We performed the present study among drug-naïve OCD patients to explore the activities and FCs of the cingulate cortexes and to further examine how they might correlate with symptom severity. The main objective was to explore whether the ACC and the PCC show different levels of resting-state activities or functional connectivities (FC) in OCD. For this purpose, ROI-based analyses were used to identify the FCs of the ACC and the PCC separately. Meanwhile, ICA was performed to detect the sub-RSNs and reveal the FC characteristics of the ACC and the PCC in these sub-RSNs.

## Materials and Methods

### Participants

This study was approved by the ethics committee of the First Affiliated Hospital of Kunming Medical College (*ClinicalTrials.gov:* NCT01298622). Patients who were seeing a doctor for the first time and who met the Diagnostic and Statistical Manual of Mental Disorders – Fourth Edition (DSM-IV) criteria for OCD (diagnosed using the Structured Clinical Interview for the DSM-IV [25]) and healthy control (HC) volunteers matched for age, sex and education were recruited for this study. All patients were recruited from the medical outpatient care unit at the Department of Psychiatry at the First Affiliated Hospital of Kunming Medical College, China. Written informed consent was obtained from all participants after the study was explained and after they went through a question-and-answer session. All participants were right-handed Han Chinese individuals between 18 and 55 years of age, and the exclusion criteria for both groups were as follows: 1) present or previous history of other psychiatric or neurological illness or serious physical disease, 2) present or previous history of alcohol or drug dependence, 3) inability to undergo an MRI scan, and 4) pregnancy. Additionally, none of the OCD patients had been treated with psychotropic drugs or psychotherapy, and their obsessive-compulsive symptoms were not due to another mental disorder or physical disease. Demographic data, including age, sex, handedness, years of education, duration of illness, and clinical symptom ratings were obtained from one experienced clinical psychiatrist prior to initiating any treatment and the MRI examination. Obsessive-compulsive symptoms were evaluated using the Yale–Brown Obsessive Compulsive Scale (YBOCS). The Hamilton Depression Rating Scale (HDRS) and the Hamilton Anxiety Scale (HAMA) were used to evaluate depressive and anxiety symptoms, respectively. Although comorbidity with anxiety or depression is common in individuals suffering from OCD, OCD is distinct from other anxiety disorders and depressions with respect to frontostriatal hyperactivity and hyper-responsivity, as well as attenuated amygdala response to disorder-independent threat stimuli [26]. Considering that comorbidity of depression and anxiety might interfere with the results of OCD, the patients with elevated depression (HDRS score>14) or anxiety (HAMA score>14) symptoms were excluded. A total of 26 OCD patients and 25 healthy controls participated in this study.

### Imaging Acquisition and Data Preprocessing

All subjects received fMRI scans with a 1.5-T clinical GE MRI scanner (Twinspeed, GE, Milwaukee, USA) using a birdcage head coil (Text S1 in File S1). After Normal T1 and T2 MRI scans were performed to exclude obvious structural abnormalities, the resting-state fMRI data were obtained using an echo-planar imaging sequence with the following parameters: repetition time (TR) = 2000 ms, echo time (TE) = 40 ms, flip angle = 90°, 24 axial slices, thickness/skip = 6/1 mm, matrix = 24×24, field of view = 240×240 mm^2^, in-plane resolution = 3.75×3.75 and scan time = 5 min 20 s.

Image preprocessing was conducted using statistical parametric mapping (SPM5; Wellcome Department of Imaging Neuroscience, London, UK; http://www.fil.ion.ucl.ac.uk/spm) software based on Matlab 7.8 (The MathWorks, Inc., Natick, MA, USA). The first 10 volumes of each functional time series were discarded, and the remaining 150 volumes of fMRI images were employed in subsequent preprocessing for slice timing using SPM5. Following the implementation of slice timing and head motion corrections, the remaining data were then spatially normalized into the MNI space and resampled to 3×3×3 mm^3^ cubic voxels. A 6-mm^3^ full-width half-maximum (FWHM) Gaussian kernel was used for spatial smoothing.

### Fractional Amplitude of Low-frequency Fluctuation (fALFF) Calculation

fALFF was calculated using the RESTing-state fMRI data analysis toolkit (http://www.restfmri.net/forum/rest_v11). First, linear trends were removed from the images to reduce low-frequency drift of the MRI equipment. The effects of the mean time course of the entire brain (i.e., global trend), white matter, and cerebrospinal fluid (CSF) were regressed out by using default masks included in the REST package [27]. Next, a fALFF map for each participant was calculated using REST software, and the data were bandpass filtered (0.01–0.08 Hz) [28]. For standardization purposes, the fALFF of each voxel was divided by the global mean fALFF value to standardize data across subjects in an analogous manner. The fALFF difference between OCD patients and controls was compared using the two-sample *t*-test in SPM5, and results were considered statistically significant at *p*<0.05 (multiple comparison correction).

### ROI-based Functional Connectivity Analysis

We also wanted to explore the functional connectivities (FC) of the regions with significantly different fALFF in OCD patients to the whole brain using a seed ROI approach. The time series of raw fMRI data for each voxel was temporally bandpass filtered (0.01–0.08 Hz). The six regions with significant fALFF changes between the OCD and the control group, as measured by the methods mentioned above, were chosen and used as six seeds for determining ROIs, especially with a focus on the regions in the ACC and the PCC areas. For each seed, a correlation analysis was carried out between each of these six ROIs with the rest of the brain in a voxel-wise manner (RESTing-state fMRI data analysis toolkit, http://www.restfmri.net/forum/rest_v11). Following this analysis, we obtained an FCMap (*R* value map) of the seed compared with all other brain voxels. Spatial smoothing was applied with a 6-mm^3^ FWHM Gaussian kernel. The group difference of FC between OCD patients and controls was compared using the two-sample *t*-test in SPM5 (*p*<0.05, corrected).

### Correlation Analysis of fALFF with Symptom Severity

To find the possible correlations between resting-state activity and the symptom severity of OCD, correlation analyses were performed between the fALFF maps and YBOCS scores. The correlation analyses were conducted using SPM5, with age and education included as covariates (*p*<0.05, corrected).

### Independent Component Analysis

We then tried to identify the functional organization of the ACC and the PCC, as well as to confirm the results from fALFF using a model-free method, the Independent Component Analysis (ICA). The ICA method can identify the resting-state sub-RSNs and find the abnormal FC of these sub-RSNs in OCD. We performed the ICA for the OCD patients and control group separately. Analyses of spatial associations were conducted using the ICA method [29] with a Group ICA Toolbox of fMRI (GIFT v1.3e; http://icatb.sourceforge.net) implemented in Matlab. After routine image normalization and spatial smoothing (6-mm^3^ FWHM), the data were analyzed separately for the patient and control groups. The results showed the same six components in both the OCD and the HC groups (Text S1 in File S1, Figure S1 in File S1). These six components were chosen as components of interest (COI) and entered into the analysis.

For each subject, the maps of the chosen COIs were then converted to *z*-value maps and entered into SPM5 for group analyses. For each component, random effects one-sample t-tests were performed separately for each group to assess the within-group integrity of the component maps. Group differences were examined using two-sample t-tests to find the abnormal FC of each sub-RSNs in OCD. The resulting statistical maps were masked with the component-specific mask of the relevant component (generated based on data from all participants) to explore the results within this network. All results had a significance threshold of *p*<0.05 (FWE corrected).

### Correlation of Component Maps with Symptom Severity

Finally, we wanted to identify the regions that were associated with OCD symptom severity in each sub-network, especially in the ACC and the PCC. For each COI, multiple regression analyses were performed to assess the relationship between the *z* value of each voxel in the individual patients’ maps and their symptom severity as assessed by the YBOCS scores. Again, analyses were controlled for age and education at threshold *p*<0.05 (small volume corrected), and the resulting statistical maps were masked with the general specific component’s mask.

## Results

### Demographic Data

In total, 26 OCD patients and 25 controls received the MRI scans. After preprocessing, the data of three patients and two controls were excluded due to a translation above 1.5 degrees or a rotation above 2 mm. The data from the remaining 23 pairs of patients and controls were entered into the analysis. There were no group differences in age, years of education or sex (8 men and 15 women in both groups) between the OCD patients and controls (Table 1). For the 23 OCD patients, the mean disease duration was 46.78±53.70 months, and the total Y-BOCS score was 31.61±6.87. The total HDRS and HAMA scores were 7.17±3.66 and 9.17±3.11, respectively.

**Table 1:** Demographic data of OCD and HC.

Variable	OCD(n = 23)	HC (n = 23)	t/χ^2^	P value
Sex (male/female)*	8/15	8/15	–	–
Age (year)	31.00(10.26)	31.65(8.85)	−0.231	0.458
Education (year)	12.04(3.78)	12.00(3.38)	0.041	0.529
Age of onset(year)	27.04(9.69)	–	–	–
Duration(month)	46.78(53.70)	–	–	–
YBOCS score	31.61(6.87)	1.87(1.29)	20.393	<0.000
HDRS score	7.17(3.66)	2.22(1.44)	4.239	<0.001
HAMA score	9.17(3.11)	1.83(1.37)	8.833	<0.001

Abbreviations: OCD, major depressive disorder; HC, healthy control; YBOCS, Yale–Brown Obsessive Compulsive Scale; HDRS Hamilton depression rating scale; HAMA, Hamilton anxiety scale. * number represents n.

### fALFF Differences between Patients and Controls

**Figure 1:**
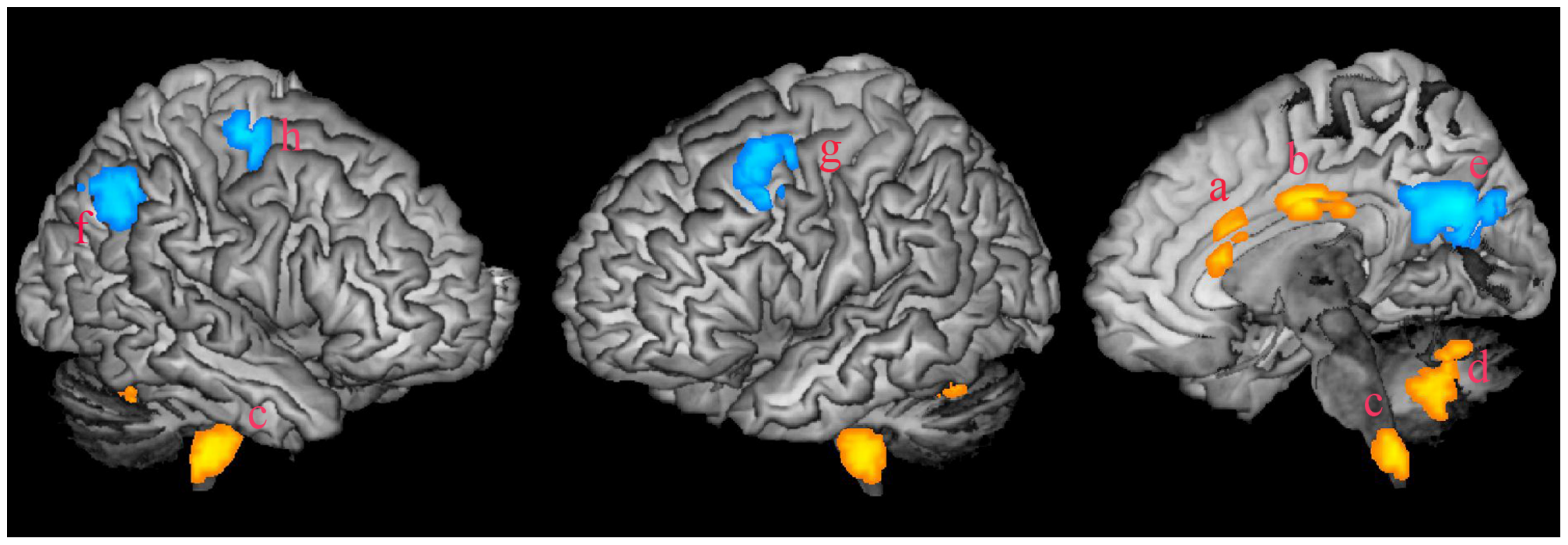
Abnormal fALFF in OCD. Four regions, mainly in the ACC, the MCC, the brainstem and the cerebellum, showed increased fALFF in subjects with OCD compared to the HC group (yellow color, small letters a to d). Four regions, located in the PCC, the IPL, the MFC and the precentral lobe separately, showed decreased fALFF in OCD (blue color, small letters e to h). Abbreviations: OCD, obsessive compulsive disorder; HC, healthy control; ACC, anterior cingulate cortex; MCC, midcingulate cortex; PCC, posterior cingulate cortex; IPL, inferior parietal lobe; MFC, middle frontal cortex.

Compared with HC, four clusters with increased fALFF were found in regions of the bilateral ACC, the middle cingulate cortex (MCC), the brainstem and the cerebellum in OCD patients (Figure 1; Table S1 in File S1). Four clusters with decreased fALFF were found in regions of the bilateral PCC, the left MFG, the right inferior parietal lobe (IPL) and the precentral lobe in OCD patients. The fALFF of the anterior and posterior parts of the cingulate gyri showed contrary abnormal changes in OCD patients.

### FC Differences between Patients and Controls

**Figure 2:**
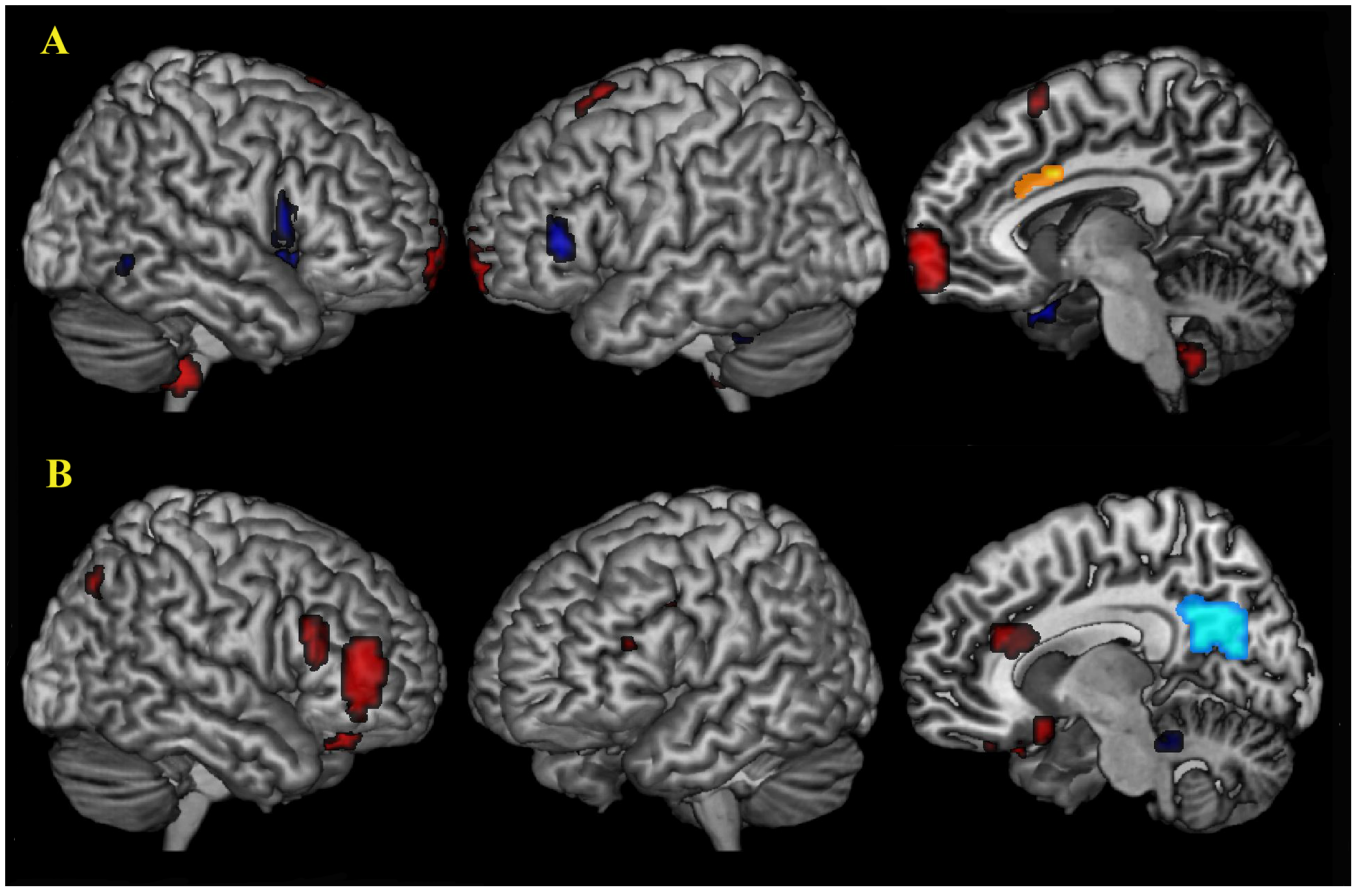
Abnormal FC of the ACC and the PCC in OCD. Compared with controls, the FC of the ACC and the PCC showed different patterns in OCD. For the ACC (yellow color), significantly increased FC with the MPFC, the brainstem, and the left SMA (red color) and decreased FC with the OFG, the DLPFC and the temporal lobe (blue color) were found in OCD group (A). For the PCC (light blue color), the results showed increased FC with the right OFG, the DLPFC and the ACC and decreased FC with the brainstem in OCD patients (B). Abbreviations: DLPFC, dorsolateral prefrontal cortex; OFG, orbital frontal gyrus; SMA, supplementary motor area.

Seven clusters identified by the above method (the ACC, the MCC, the brainstem, the PCC, the MFG, the IPL, and the precentral lobe) were chosen as ROIs. Using these clusters as ROIs, the functional connectivity of each ROI with whole brain in OCD patients and controls was calculated separately. Compared with controls, the FCs of the ACC and the PCC also showed different patterns in OCD. For the ACC, FC significantly increased with the PFC, the brainstem, and the left supplementary motor area (SMA) but decreased with the OFG and the temporal lobe (especially in the right insula, the inferior temporal lobe (ITG), and the superior temporal lobe (STG)) in the OCD group. For the PCC, the results showed increased FC with the right brain, the ACC, the DLPFC and the OFG but decreased FC with the midbrain in OCD patients (Figure 2; Table S2 in File S1; Figure S2 in File S1).

Compared with controls, patients with OCD also showed abnormal patterns of FC in five other ROIs (the MCC, the brainstem, the MFG, the IPL and the precentral lobe; Figure S2 in File S1). For example, the precentral lobe showed decreased FC with the vast area of the frontal lobe. The brainstem showed increased FC with the frontal lobe but decreased FC with the putamen.

### Correlation of fALFF with Symptom Severity

The results of the correlation analyses revealed that the YBOCS scores and fALFF were positively correlated in the right MFG region (D) and the cerebellum (A). However, a negative correlation between YBOCS scores and the fALFF was found in the PCC region (B) and the right superior temporal lobe (Table S3 in File S1; Figure S3 in File S1).

### ICA Results for the Two Groups

Using ICA methods, we identified the same six components in the OCD patient and control groups separately (Figure S1 in File S1). These six components represented 6 sub-networks of the resting state. Component 1 (IC1) included the network consisting of the posterior cingulate/precuneus, the medial frontal regions and the bilateral parietal/temporal regions and is known as the default mode network (DMN) [24], which is suggested to be involved in episodic memory and self-projection. Component 2 (IC2) was the network consisting of the bilateral superior temporal region, the insula region and the hippocampi, which play important roles in memory (MN). Component 3 (IC3) was the sensorimotor network, including the SMA and the pre/post central regions (SMN). Component 4 (IC4) was composed of the ventromedial prefrontal cortex (VMPFC), the medial orbital frontal cortex (OFG), the gyrus rectus, and the pregenual ACC and is known as the self-referential network (SRN). Component 5 (IC5) was the visual network (VN), which included the inferior, middle and superior OFG, the temporal-occipital regions, and superior parietal gyrus. Component 6 (IC6), or the lateral parietal-frontal network, included the dorsal lateral prefrontal cortices and posterior parietal cortices and is also known as the central executive network (CEN) [24]. Consistent with our hypotheses, the ACC and the PCC were located in different components (the ACC in IC4 and the PCC in IC1).

**Figure 3:**
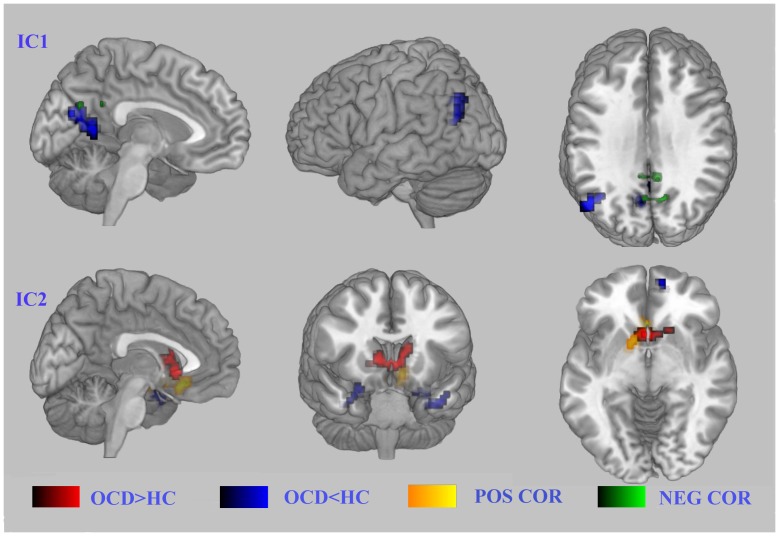
Abnormal FC of the ACC and the PCC correlated with symptom severity of OCD. Panel A shows the networks that comprise the PCC, while panel B shows the networks that comprise the ACC. A negative correlation between YBOCS scores and FC magnitudes of the precuneus/PCC was found (A); in contrast, a positive correlation between YBOCS scores and FC magnitudes was found in the ACC (B). Abbreviations: YBOCS, Yale–Brown Obsessive Compulsive Scale.

Voxel-wise, two-sample t-tests revealed significant differences in the regional FC strengths of these ICs between the two groups (Table S4 in File S1; Figure S4 in File S1). Compared with the HC, the OCD patients showed obviously decreased FC in IC1 (DMN, mainly in the precuneus and the PCC). For IC4, increased FC was located mainly in the thalamus/ACC, with decreased FC located mainly in the PFC (Figure 3). Significant FC differences between groups in the ACC and the PCC were found separately.

### Correlations of the RSN Connectivity Maps with Symptom Severity

The correlations between the component map and total YBOCS scores were explored (Table S5 in File S1; Figure S4 in File S1). Positive correlations with YBOCS scores were found for IC4, while negative correlations were found for IC1. For IC1 (DMN), a negative correlation between the YBOCS scores and the FC magnitude of the precuneus/PCC was found (Figure 3). For IC4 (SRN), a positive correlation between YBOCS scores and the ACC was found. These regions with abnormal FC overlapped with the regions that correlated with symptom severity. The PCC/precuneus regions with a decreased FC were negatively correlated with the total YBOCS scores. The pregenual ACC/thalamus regions with increased FC were positively correlated with the total YBOCS scores. However, the right lateral frontal lobe regions with decreased FC were positively correlated with the total YBOCS score (Figure 3).

There were some other correlations with YBOCS scores for the other 4 ICs, including a positive correlation of MTL in IC2, a negative correlation of SMA in IC3, a negative correlation of occipital lobe in IC5, and a positive correlation of the middle frontal gyrus in IC6 (Table S5 in File S1).

## Discussion

In this study, we found opposing activities for the ACC and the PCC in OCD. The patients with OCD showed significantly increased activity in the ACC and the MCC but decreased fALFF in the PCC compared to the controls. On the other hand, the FC of ACC and PCC showed distinct patterns. FC increased from the anterior portion of the cingulate (ACC/MCC) to the SFG/MFG, the midbrain and the SMA but decreased to the OFG, the IFG and the temporal lobe. In contrast, FC increased from the PCC to the OFG and the DLPFC but decreased to the midbrain in the OCD patients. These results may suggest an imbalanced function of the ACC and the PCC in drug-naïve OCD patients. Some regions with abnormal FC, especially the ACC and the PCC, were correlated with symptom severity. The resting-state activity of the PCC was even correlated with symptom severity. Both abnormal activity and FC, especially in the PCC, were correlated with symptom severity in our results, supporting the role of resting-state functional activity of the cingulate in the core deficits of OCD.

Different portions of the cingulate gyrus have distinct fiber connections and functions [30]. ICA analysis revealed that the ACC and the PCC were located in different sub-networks and showed opposite correlation with symptom severity. This result confirms that these regions might have different functional organizations and execute different functions in OCD. The ACC/MCC is vital to response selection, whereas the PCC plays a role in personal self-relevance assessment and episodic memory processes [31]. The ACC is an important part of the CSPT circuit, which is associated with excessive activity in OCD patients both at rest and during cognitive tasks [4], [32]. The most basic ACC theory states that the ACC is involved with error detection and conflict monitoring. Paus et al. has suggested that the ACC is a region where regulatory and executive processes interact [33]. In this model, the outputs of cognitive processing performed in the prefrontal cortex are combined in the ACC with representations of emotional state to enable appropriate behavioral responses to internal or environmental events. Abnormal increased activity of the ACC may be related to excessive error detection and conflict monitoring in OCD. A recent study has found that the FC between subgenual ACC and the ventral PFC is related with a sad mood in OCD [34]. However, scientists have proposed that the PCC plays a role in human awareness and memory retrieval. Here, we found that the PCC showed decreased activity in OCD patients. The PCC is centrally located along the default mode network (DMN) that controls the brain when the body and mind are at rest. Brain scans have shown that the nerves of the posterior cingulate cease firing while a person performs a task, but these nerves reactivate when the task is completed. The DMN is thought to be related to self-awareness and control. Decreased activity of the PCC may result in lower self-control in OCD patients. The tonic hyperactivity of the ACC but hypoactivity of the PCC may reflect the pathophysiology of high error detection but low self-control in OCD patients. Additionally, executive functions are carried out largely by the prefrontal areas of the frontal lobe. The frontal lobe has been emphasized in OCD research. The PFC can guide the selection of goal-directed action sequences [35]. Dysfunction in the PFC is related to the inability of patients to exercise control over compulsive cognitions, especially intrusive, compulsive thoughts and their respective motor reactions [36]. We identified distinct FC patterns of the ACC and the PCC input to the vast frontal areas. The ACC showed increased FC to the DMPFC but decreased FC to the DLPFC. These results might suggest the relatively strong control of the DMPFC but weak control of the DLPFC on the ACC. The DMPFC is critical for monitoring the addition versus subtraction of actions for counterfactual reasoning [35]. In contrast, the DLPFC supports complex processes operating on information (spatial and non-spatial) being maintained in working memory, such as monitoring, manipulation, and higher-level planning. We also found increased DLPFC-PCC connectivity. These interaction effects may reflect compensatory manipulation and increased tonic arousal at rest. Our results, in combination with those of previous studies, support a conflict-control deficit characterized by tonic sensitivity of conflict control and suppression of internal monitoring in OCD.

In our results, the ACC showed decreased FC to the OFC, while the PCC showed increased FC to the OFC. Connectivity of the sgACC and the PCC also correlated with symptom severity in OCD patients. The OFC is a prefrontal region that is involved in the cognitive process of decision-making. Because of its functions in emotions and reward processing, the OFC is considered a part of the limbic system. In particular, the human OFC is thought to regulate planning behavior associated with sensitivity to reward and punishment [4]. Disorders of executive function and impulse control, such as OCD, may be affected by OFC circuitry dysregulation. Walton et al. demonstrated the reciprocal role of the OFC and the sgACC in monitoring behavioral outcomes [37]. Dissociation of value computations in OFC and ACC neurons has also been reported [38]. OFC neurons dynamically evaluate current choices relative to recent choice values, whereas ACC neurons encode choice predictions and prediction errors. Our results strongly support an imbalance within a cingulate-frontal network, which might reflect disparity in error detection and self-control in OCD patients.

Six sub-networks were identified as having resting-state activity that closely resembled those reported in previous studies [22]. These six sub-networks were related to important tonic brain functions when in a resting state, such as self-monitoring (DMN, IC1), self-reorientation (SRN, IC4), memory (MN, IC2), sense and motor control (SMN, IC3), and executive function (CEN, IC6). Although the six sub-networks were spatially similar across patients and controls, the regional FC strength of these sub-networks showed significant differences between the groups, supporting the vast abnormal resting-state activity in OCD patients, thought to result in the loss of self-control. A component including the occipital lobe and the cerebellum was implicated among the healthy controls but not among the subjects with OCD, implying relatively weak activities or a lack of connection in these two areas in OCD patients.

Abnormal activity in other structures, including the pre-central lobe, the SMA, the midbrain, and the cerebellum, was also found in our results. The pre-central portion showed decreased activity and FC to the OFG and the IFG. Because the pre-central portion is related to sensory control, abnormal activity or FC to the PFC may destroy the PFC’s control over sensory integrity in OCD. The SMA plays a crucial role in the generation of memory sequences that fit into a precise timing plan [39]. Thus, the abnormal activity of the SMA may serve as the basis of compulsive behavior. We found especially strong FC between the ACC and the SMA, suggesting a strong synchrony between these two regions at a rest-state; this may be the pathological basis of compulsive behavior of OCD. A few studies have reported abnormal midbrain structure and function in OCD, such as enlarged grey matter volume [40] and higher glucose metabolism [41]. Nuclei, which emanate neurotransmitters such as serotonin, dopamine, and norepinephrine, are all located in the midbrain. Functional imaging studies found reduced availabilities [42] or binding [43] of the midbrain and brainstem serotonin transporters in OCD. The increased FC between the midbrain and the putamen in the present study might imply abnormal tonic monoaminergic transduction between the midbrain and thalamus in OCD patients.

Our results strongly support that OCD is a complex disease that features abnormal and opposing activities and differential connectivities of the ACC and the PCC in the resting state. These imbalanced activities and connectivities in the ACC and the PCC, or misalignment of their input-output properties, are part of the pathology of OCD. Thus, the future direction of treatment may focus on how to adjust both hyper- and hypotonic neural activities in patients. However, due to the relatively small sample size and because most patients showed symptoms of both obsessive thoughts and behaviors, we could not identify the specific networks underlying OCD symptoms. Future studies that focus on the different symptoms or subtypes of OCD may help to further the understanding of the pathophysiology of OCD.

## Supporting Information

File S1 **Text S1. Table S1, Abnormal fALFF in OCD. Table S2, FC difference of ACC and PCC between OCD and HC. Table S3, Results of Correlation analysis between fALFF and YBOCS in OCD. Table S4, FC difference of six components between OCD and HC. Table S5, Regions correlated with the total score of YBOCS in six ICs. Figure S1, Six ICs in OCD and HC. Figure S2, Abnormal FC for 7 ROIs in OCD. Figure S3, Correlation of fALFF and score of YBOCS. Figure S4, Abnormal FC in OCD and the correlation with the symptom severity in six sub-RSNs.**
(DOC)
