# Supplementary material for: Abnormal Resting-State Activities and Functional Connectivities of the Anterior and the Posterior Cortexes in Medication-Naïve Patients with Obsessive-Compulsive Disorder
Source: PLoS One. 2013 Jun 28;8(6):e67478. doi: 10.1371/journal.pone.0067478 (PMC3696097; doi:10.1371/journal.pone.0067478)
Supplement: File S1 — Text S1. Table S1, Abnormal fALFF in OCD. Table S2, FC difference of ACC and PCC between OCD and HC. Table S3, Results of Correlation analysis between fALFF and YBOCS in OCD. Table S4, FC difference of six components between OCD and HC. Table S5, Regions correlated with the total score of YBOCS in six ICs. Figure S1, Six ICs in OCD and HC. Figure S2, Abnormal FC for 7 ROIs in OCD. Figure S3, Correlation of fALFF and score of YBOCS. Figure S4, Abnormal FC in OCD and the correlation with the symptom severity in six sub-RSNs. (DOC) [file pone.0067478.s001.doc]

**Supplemental Methods and Results**

**Text S1.**

**Imaging Acquisition and Data Preprocessing**

One experienced neuroradiologist performed all the image acquisitions with a 1.5-T clinical GE MRI scanner (Twinspeed, GE, Milwaukee, USA). All subjects were placed in a birdcage head coil and fitted with foam padding to minimize head motion. During the scanning process, noise and light interference were blocked with rubber earplugs and a black eye shield. To maintain resting-state (3, 18), all subjects were instructed to hold still, keep their eyes closed and to have as few thoughts as possible without falling asleep. Normal T1 and T2 MRI scans were performed first to exclude obvious structural abnormalities. Then resting-state fMRI data were obtained using an echo-planar imaging sequence with the following parameters: repetition time (TR) =2000 ms, echo time (TE) =40 ms, flip angle=90°, 24 axial slices, thickness/skip=6/1 mm, matrix=24×24, field of view=240×240 mm^2^, in-plane resolution=3.75×3.75, scan time=5 min 20 s. The volume of each brain was comprised of 24 axial slices and each functional run contained 160 image volumes.

**Data Preprocessing**

Image preprocessing was conducted using statistical parametric mapping (SPM5, Wellcome Department of Imaging Neuroscience, London, UK. http://www.fil.ion.ucl.ac.uk/spm) software based on Matlab 7.8 (The MathWorks, Inc. Natick, MA, USA). DICOM images were transformed to ANALYSE format using MRIcroN software (http://www.sph.sc.edu/comd/rorden/mricron). The first 10 volumes of functional time series were discarded because of possible instability of the initial signal. The remaining 150 volumes of fMRI images were employed in subsequent preprocessing for slice timing using SPM5. After slice timing, head motion corrections were done, and the data for which the head motion was above 2 mm for translation and 1.5° for rotation were discarded. The remaining data were then spatially normalized into the MNI space and resampled to 3×3×3 mm^3^ cubic voxels. Finally, spatial smoothing was conducted with a 6-mm^3^ full-width half-maximum (FWHM) Gaussian kernel.

**Identifying components of OCD and HC group using ICA**

First, principal component analysis was used to estimate the number of components in the combined data sets of controls and OCD patients, resulting in 23 control components and 21 patient components.

A systematic process was used to identify the components for further analysis. The association of each component’s spatial map with a priori probabilistic maps of gray matter, white matter, and cerebral spinal fluid within standardized brain space (MNI templates provided in SPM5) helped to identify those components whose patterns of correlated signal change largely consisted of gray matter versus non-gray matter. Components with high correlation to a priori localized cerebrospinal fluid (CSF) or white matter or components with low correlation to gray matter suggested they may be artifacts instead of signs of hemodynamic change. In the OCD group, 6 components were discarded because they had less than a 0.025 R^2^ association with gray matter. Two components were related to the spatial distribution of white matter, and 7 components were related to the spatial distribution of CSF because their R2 association with WM/CSF was greater than 0.025. These 9 components were also discarded. Visual inspection of the discarded components suggested that they represented eyeball movements, head motion, and cardiac-induced pulsatile artifacts at the base of the brain and were considered to reflect patterns unrelated to neural activity. Fifteen control components were discarded using the same methods (two for correlation with WM, 12 for correlation with CSF and one for low correlation with GM). After the removal of discarded components from the raw data, 6 patient components and 7 control components remained, and new ICAs were performed. There were 6 same components in both OCD and HC in addition to a component that included the occipital lobe and cerebellum in HC group only. These six components were then compared between groups.

**Statistics for components**

For each subject, the chosen six components were then converted to z values. To create a sample-specific component map, individual maps of all subjects regardless of group were entered into random effect one-sample t-tests in SPM5 for each component, and the significance threshold was set at p<0.01, corrected for family-wise error(FWE). These maps were used as a mask for group analyses within the corresponding component. Thus, the results are not biased by the components maps from healthy participants only. Individual DMN component GIFT maps were entered into SPM5 for group analyses. The z values in these individual maps represent the fit of a specific voxel BOLD time course to the group averaged of each component's time course. For each component, random effects one-sample t-tests were performed for each group separately to assess the within-group integrity of the component maps. Random effects two-sample t-tests examined group differences. The resulting statistical maps were masked with the general map of the study-specific relevant component (generated based on data from all participants) to explore the results within only this network. The significance threshold for all results was set at p<0.05, corrected (FWE).

**Table S1. Abnormal fALFF in OCD**

| **Brain region** | **Hemisphere** | **Cluster size**  **(voxels)** | ***t*** | **MNI**  **(x y z)** | | |
| --- | --- | --- | --- | --- | --- | --- |
| ***OCD>HC*** |  |  |  |  | | |
| **ACC^a^** | **R,L** | **37** | **3.26** | **0** | **21** | **30** |
| **MCC^b^** | **R** | **42** | **3.89** | **3** | **-6** | **33** |
| **Brainstem^c^** | **R** | **50** | **3.77** | **3** | **-39** | **-57** |
| **Cerebellum^d^** | **R** | **33** | **4.16** | **21** | **-45** | **-57** |
|  | **L** | **71** | **3.47** | **-12** | **-45** | **-30** |
| ***OCD<HC*** |  |  |  |  |  |  |
| **PCC^e^** | **R,L** | **189** | **4.65**  **4.21** | **9**  **-9**  **0** | **-66**  **-69**  **-75** | **27**  **21**  **27** |
| **Inferior parietal lobe^f^** | **R** | **63** | **3.96** | **39** | **-54** | **45** |
| **MFG^g^** | **L** | **64** | **3.93** | **-24** | **6** | **57** |
| **Precentral gyrus^h^** | **R** | **39** | **3.80** | **33** | **-15** | **63** |

Abbreviations: OCD, obsessive–compulsive disorder ; HC, healthy control; R, right; L, left; ACC, anterior cingulate cotex; MCC, midcingulate cotex; PCC, posterior cingulate cotex; MFG, meddle frontal gyrus;

**Table S2. FC difference of ACC and PCC between OCD and HC**

| **ROIs** | **Hemisphere** | **Brain region** | **Cluster size**  **(voxels)** | ***t*** | **MNI**  **(x y z)** | | |
| --- | --- | --- | --- | --- | --- | --- | --- |
| **ACC** |  |  |  |  |  |  |  |
| **OCD>HC** | **L** | **Medial Frontal Gyrus** | **185** | **4.59** | **-9** | **63** | **0** |
|  | **R** | **Midbrain** | **107** | **4.35** | **6** | **-39** | **-54** |
|  | **L** | **Supp_Motor_Area_L** | **22** | **3.34** | **-12** | **15** | **69** |
| **OCD<HC** | **R** | **Insula_R** | **82** | **4.54** | **33** | **12** | **-6** |
|  | **R** | **Superior Temporal Gyrus** | **23** | **3.78** | **24** | **15** | **-30** |
|  | **L** | **Fusiform_L** | **24** | **3.65** | **-39** | **-6** | **-18** |
|  | **L** | **Inferior Frontal Gyrus** | **43** | **3.60** | **-51** | **33** | **9** |
|  | **R** | **Temporal_Inf_R** | **22** | **3.40** | **45** | **-57** | **-12** |
| **PCC** |  |  |  |  |  |  |  |
| **OCD<HC** | **R** | **Midbrain** | **26** | **3.70** | **3** | **-36** | **-24** |
| **OCD>HC** | **R** | **Frontal_Inf_Orb_R** | **272** | **4.88** | **24** | **39** | **-15** |
|  | **R** | **Anterior Cingulate** | **33** | **4.67** | **6** | **24** | **18** |
|  | **R** | **Inferior Frontal Gyrus** | **37** | **3.89** | **51** | **27** | **18** |
|  | **L** | **Frontal_Inf_Tri_L** | **22** | **3.67** | **-39** | **27** | **18** |
|  | **L** | **Middle Frontal Gyrus** | **22** | **3.65** | **-39** | **6** | **33** |
|  | **R** | **Olfactory_R** | **28** | **3.54** | **9** | **15** | **-18** |
|  | **R** | **Angular_R** | **22** | **3.43** | **39** | **-66** | **42** |

Abbreviations: OCD, obsessive–compulsive disorder ; HC, healthy control; R, right; L, left; ACC, anterior cingulate cotex; PCC, posterior cingulate cotex.

**Table S3. Results of Correlation analysis between fALFF and YBOCS in OCD**

| **Brain region** | **Hemisphere** | **Cluster size**  **(voxels)** | ***t*** | **MNI**  **(x y z)** | | |
| --- | --- | --- | --- | --- | --- | --- |
| **Positive correlation** |  |  |  |  | | |
| **Cerebellum** | **L** | **24** | **5.04** | **-6** | **-57** | **-18** |
| **Meddle frontal gyrus** | **R** | **21** | **3.94** | **45** | **27** | **33** |
| **Negative correlation** |  |  |  |  |  |  |
| **PCC** | **R** | **39** | **2.89** | **6** | **-81** | **12** |
| **Superior temporal lobe** | **R** | **64** | **2.88** | **48** | **-42** | **15** |

Abbreviations: OCD, obsessive–compulsive disorder ; R, right; L, left; PCC, posterior cingulate cotex; YBOCS,Yale–Brown Obsessive Compulsive Scale.

**Table S4. FC difference of six components between OCD and HC.**

|  | **Hemisphere** | **Brain region** | **Cluster size**  **(voxels)** | ***T*** | **MNI**  **(x y z)** | | |
| --- | --- | --- | --- | --- | --- | --- | --- |
| **IC1** |  |  |  |  |  | | |
| **OCD<HC** | **L** | **Precuneus** | **81** | **3.33** | **-9** | **-57** | **9** |
|  | **L** | **Angular Gyrus** | **46** | **3.32** | **-48** | **-69** | **36** |
| **IC2** |  |  |  |  |  |  |  |
| **OCD<HC** | **L** | **Superior Temporal Gyrus** | **54** | **5.02** | **-42** | **-15** | **3** |
|  | **R** | **Superior Temporal Gyrus** | **137** | **4.85** | **48** | **-33** | **15** |
| **IC3** |  |  |  |  |  |  |  |
| **OCD>HC** | **L** | **Insula** | **48** | **4.79** | **-36** | **21** | **-3** |
|  | **R** | **Superior Temporal Gyrus** | **130** | **4.78** | **48** | **-9** | **-3** |
|  | **L** | **Superior Temporal Gyrus** | **80** | **3.84** | **-45** | **-9** | **-3** |
|  | **R** | **Inferior Frontal Gyrus** | **79** | **4.62** | **39** | **21** | **-9** |
|  | **R** | **Inferior Parietal Lobule** | **40** | **3.93** | **54** | **-30** | **33** |
| **OCD<HC** | **L** | **Supp_Motor_Area** | **74** | **4.96** | **-6** | **-3** | **60** |
|  | **R** | **Pre/Postcentral Gyrus** | **340** | **4.90** | **63** | **-3** | **18** |
|  | **L** | **Pre/Postcentral Gyrus** | **318** | **4.87** | **-57** | **-15** | **42** |
| **IC4** |  |  |  |  |  |  |  |
| **OCD>HC** | **L** | **Anterior cingulate/ Caudate** | **68** | **4.10** | **-3** | **15** | **0** |
| **OCD<HC** | **R** | **Medial Frontal Gyrus** | **33** | **3.32** | **3** | **57** | **9** |
|  | **L** | **Superior Temporal Gyrus** | **24** | **3.54** | **39** | **9** | **-33** |
|  | **R** | **Superior Temporal Gyrus** | **46** | **3.24** | **-39** | **15** | **-33** |
| **IC5** |  |  |  |  |  |  |  |
| **OCD>HC** | **R** | **Occipital Lobe/ Cuneus** | **325** | **5.44** | **12** | **-99** | **18** |
| **OCD<HC** | **R** | **Posterior Cingulate** | **213** |  | **15** | **-63** | **21** |
|  | **R** | **Fusiform** | **26** | **3.39** | **27** | **-42** | **-15** |
| **IC6** |  |  |  |  |  |  |  |
| **OCD>HC** |  | **Superior Frontal Gyrus** | **58** | **5.85** | **0** | **12** | **66** |
|  | **L** | **Middle Temporal Gyrus** | **178** | **4.84** | **-63** | **-24** | **-6** |
|  | **L** | **Middle Temporal Gyrus** | **85** | **4.37** | **-6** | **45** | **45** |
|  | **R** | **Middle Temporal Gyrus** | **124** | **3.71** | **48** | **-30** | **-6** |
|  | **L** | **Inferior Frontal Gyrus** | **56** | **3.65** | **-51** | **12** | **-6** |
|  | **R** | **Inferior Frontal Gyrus** | **43** | **3.48** | **24** | **-75** | **-27** |
|  | **L** | **Temporal_Pole_Sup** | **20** | **3.44** | **-45** | **21** | **-21** |
| **OCD<HC** | **R** | **Inferior Frontal Gyrus** | **60** | **3.82** | **48** | **45** | **0** |
|  | **R** | **Inferior Parietal Lobule** | **27** | **3.66** | **69** | **-30** | **33** |

Abbreviations: OCD, obsessive–compulsive disorder ; HC, healthy control; R, right; L, left; IC1-6,independent component 1-6.

**Table S5. Regions correlated with the total score of YBOCS in six ICs.**

|  | **Positive correlation** | | | | | **Negative correlation** | | | | |
| --- | --- | --- | --- | --- | --- | --- | --- | --- | --- | --- |
|  | **Brain region** | **Cluster size**  **(voxels)** | **MNI**  **(x y z)** | | | **Brain region** | **Cluster size**  **(voxels)** | **MNI**  **(x y z)** | | |
| **IC1** |  |  |  |  |  | **Precuneus** | **35** | **9** | **-66** | **30** |
|  |  |  |  |  |  | **Post Cingulate** | **34** | **-6** | **-48** | **27** |
| **IC2** | **Middle Temporal Gyrus** | **28** | **-51** | **-12** | **-12** |  |  |  |  |  |
| **IC3** |  |  |  |  |  | **SMA** | **39** | **0** | **-21** | **60** |
| **IC4** | **Anterior Cingulate*** | **55** | **-6** | **21** | **-15** |  |  |  |  |  |
| **IC5** |  |  |  |  |  | **Occipital Lobe*** | **50** | **21** | **-54** | **-9** |
| **IC6** | **Middle Frontal Gyrus** | **30** | **42** | **39** | **-6** |  |  |  |  |  |

**P*<0.05, otherwise *P*<0.01. Abbreviations: OCD, obsessive–compulsive disorder ; HC, healthy control; R, right; L, left; SMA, supplement motor area; IC 1-6,independent component 1-6.


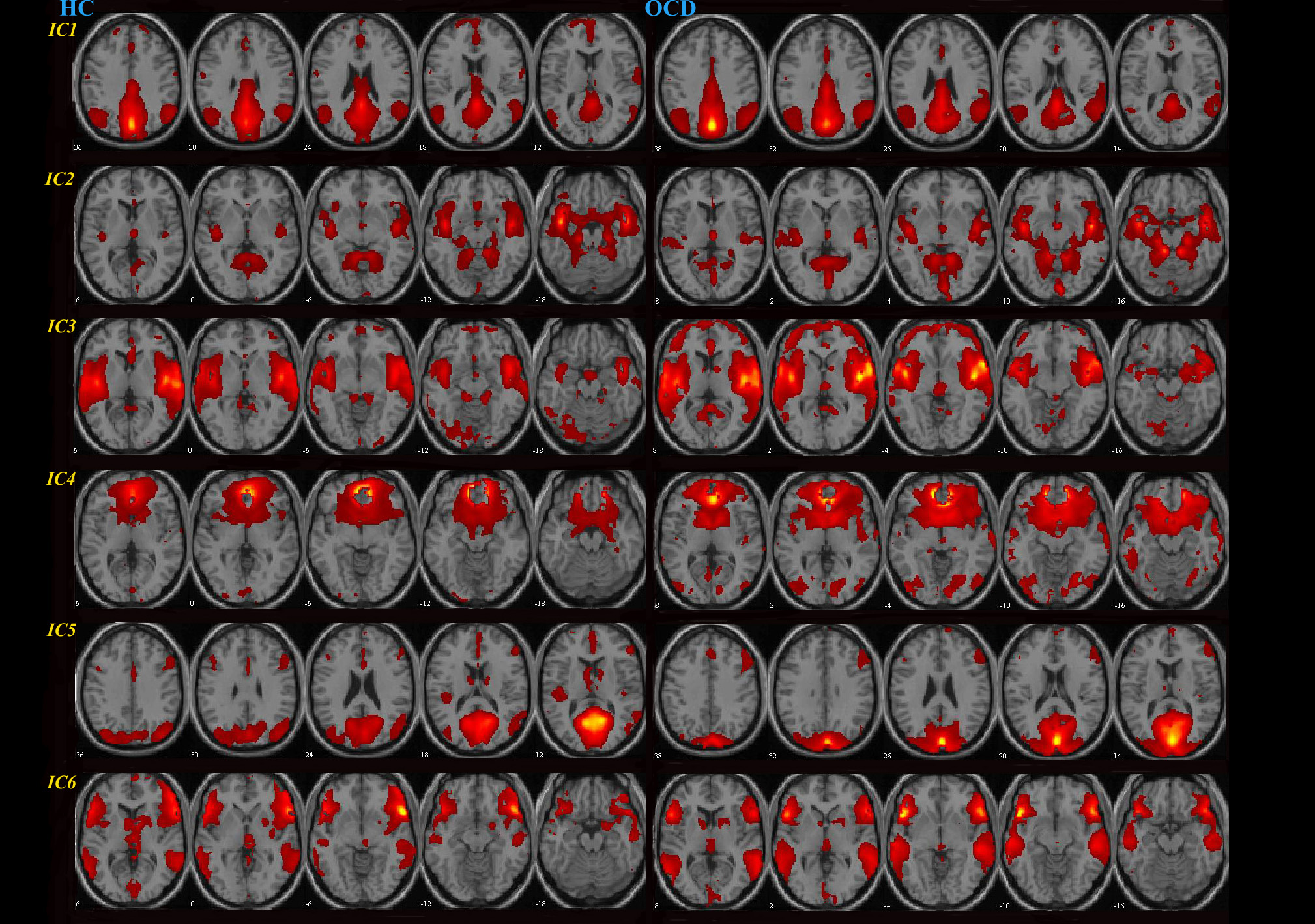


**Figure S1. Six ICs in OCD and HC.** Using ICA methods, six ICs were identified both in OCD and HC groups. These six components represented 6 networks. Component 1 (IC1), which includes the network consisting of the posterior cingulate/precuneus, medial frontal regions and bilateral parietal/temporal regions, was known as the default mode network (DMN). Component 2 (IC2) was the network consisting of the bilateral superior temporal region, insula region and hippocampi, which play important roles in memory (MN). Component 3 (IC3) was the sensorimotor network, including the SMA and pre/post central regions (SMN). Component 4 (IC4) was comprised of the ventromedial prefrontal cortex (VMPFC), medial orbital frontal cortex (OFG), gyrus rectus, and pregenual ACC, also known as the self-referential network (SRN). Component 5 (IC5) is the visual network (VN) network, which includes the inferior, middle and superior OFG, the temporal-occipital regions, and superior parietal gyrus. Component 6 (IC6) or the lateral parietal-frontal network, includes the dorsal lateral prefrontal cortices and posterior parietal cortices, is also known as the central executive network (CEN).


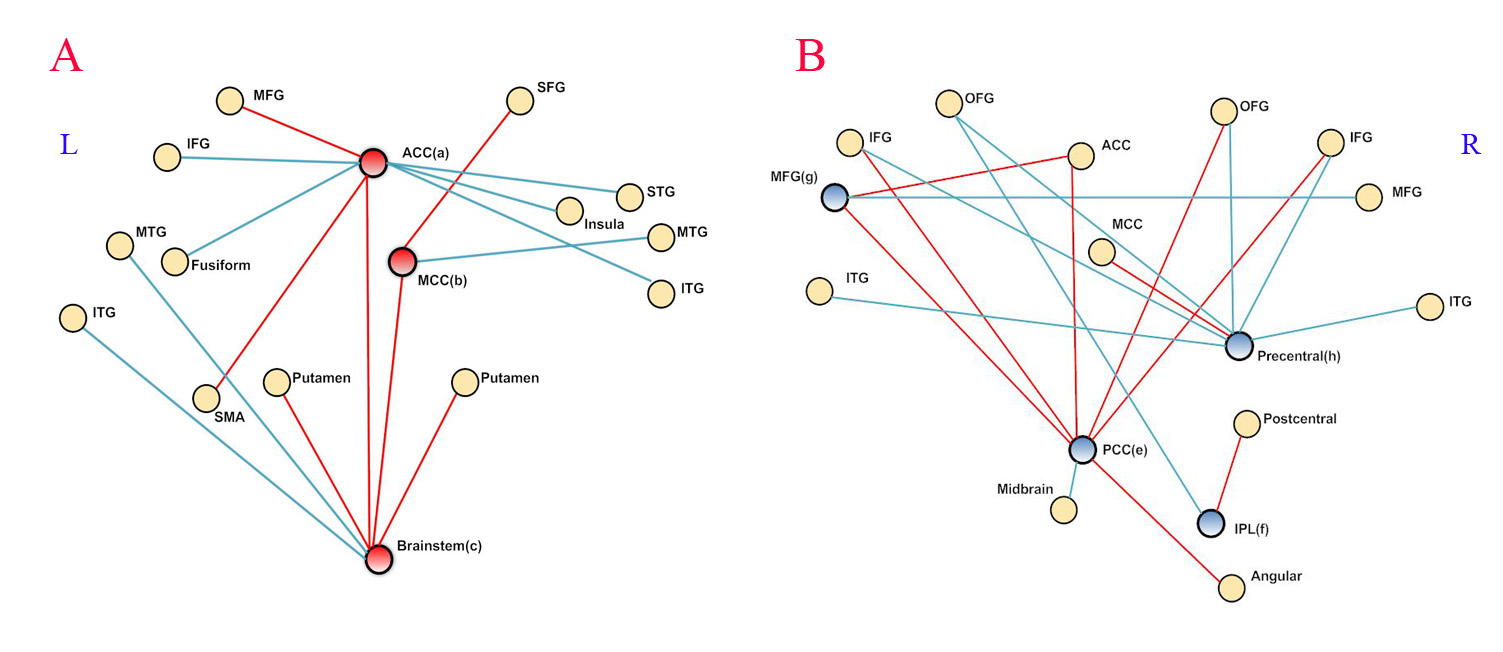


**Figure S2. Abnormal FC for 7 ROIs in OCD.**Regions with increased fALFF located in ACC, MCC, brainstem and cerebellum (red color). Regions with decreased fALFF located in MFG, PCC, IPL and precentral lobe (blue color). Compared with controls, patients showed different pattern of FC of these seven ROIs (ACC, MCC, brainstem, PCC, MFG, IPL and precentral lobe). Red lines represent the increased FC in OCD. Blue lines represent the decreased FC in OCD (Panel B and C). Abbreviations: ACC, anterior cingulate cotex; MCC, middle cingulate cotex; PCC, posterior cingulate cotex; MFG, meddle frontal gyrus; IPL, OFG, orbital frontal gyrus; inferior parietal lobe; IFG, inferior frontal gyrus; MFG, middle frontal gyrus; SFG, superior frontal gyrus; ITG, inferior temporal lobe; MTG, middle temporal gyrus; STG, superior frontal gyrus; SMA, supplementary motor area; R right; L, left.

**
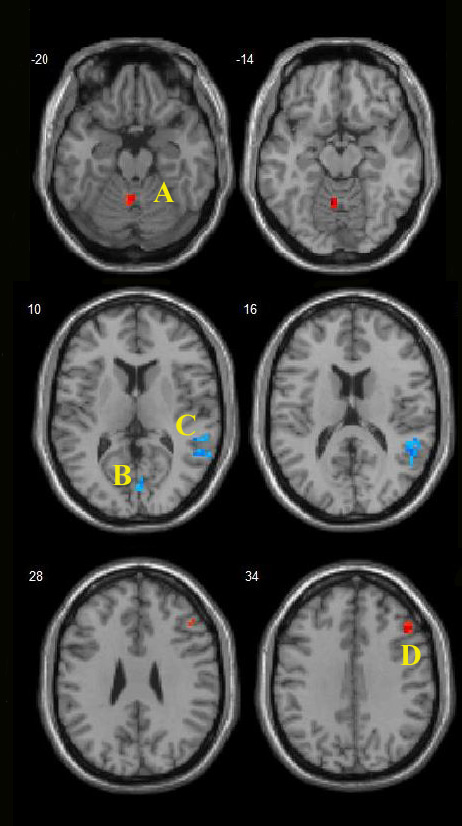
**

**Figure S3. Correlation of fALFF and score of YBOCS.** The results of correlation analyses revealed that the score of YBOCS and fALFF were positively correlated in the region of right MFG (D) and cerebellum(A). However, the negative correlation between YBOCS and fALFF was found in the region of PCC (B) and right superior temporal gyrus (C). Abbreviations: PCC, posterior cingulate cotex; MFG, meddle frontal gyrus.


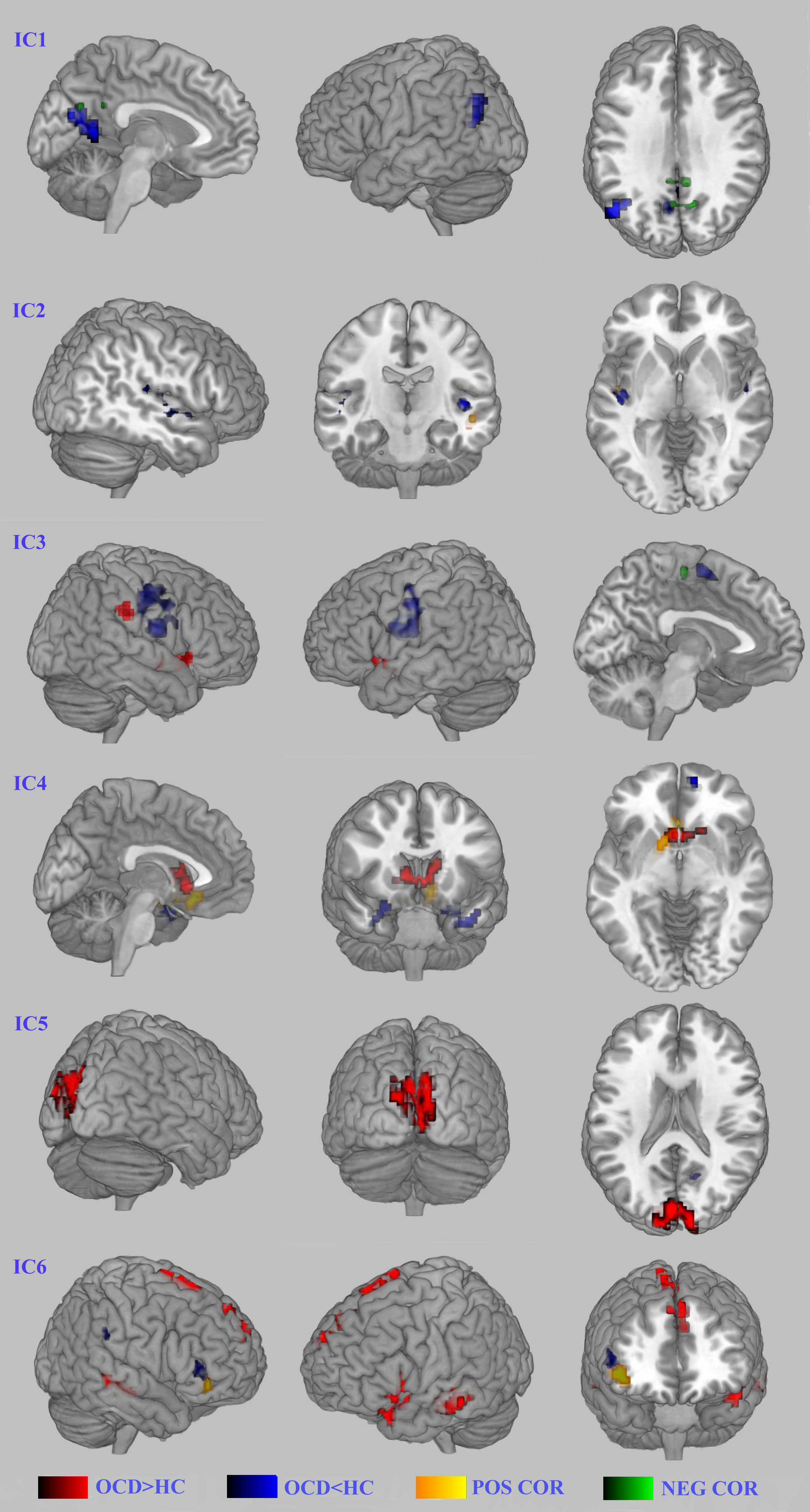


**Figure S4. Abnormal FC in OCD and the correlation with the symptom severity in six sub-RSNs.** Regions with increased FC (red color) or decreased FC (blue color) in OCD were found in 6 ICs(or sub-RSNs). Some regions in these 6 sub-RSNs were correlated with the symptom severity of OCD (positive correlation: yellow color; negative correlation: green color). IC1 and IC4 were the same ICs showed in figure 3. Abbreviations: OCD, major depressive disorder; HC, healthy control; IC1-6, independent component 1-6.
